# Albendazole Detection at a Nanomolar Level Through a Fabry–Pérot Interferometer Realized via Molecularly Imprinted Polymers

**DOI:** 10.3390/s25206456

**Published:** 2025-10-18

**Authors:** Ines Tavoletta, Ricardo Oliveira, Filipa Sequeira, Catarina Cardoso Novo, Luigi Zeni, Giancarla Alberti, Nunzio Cennamo, Rogerio Nunes Nogueira

**Affiliations:** 1 Department of Engineering, University of Campania Luigi Vanvitelli, Via Roma 29, 81031 Aversa, Italy; ines.tavoletta@unicampania.it (I.T.); luigi.zeni@unicampania.it (L.Z.); 2 Instituto de Telecomunicações, University of Aveiro, Campus Universitário de Santiago, 3810-193 Aveiro, Portugal; oliveiraricas@av.it.pt (R.O.); fsequeira@av.it.pt (F.S.); catarinacnovo@av.it.pt (C.C.N.); rnogueira@av.it.pt (R.N.N.); 3 Department of Chemistry, University of Pavia, Via Taramelli 12, 27100 Pavia, Italy; giancarla.alberti@unipv.it

**Keywords:** Fabry–Pérot interferometer (PFI) probe, molecularly imprinted polymers (MIPs), single-mode fiber (SMF), albendazole (ABZ) detection, optical-chemical sensor

## Abstract

Albendazole (ABZ) is a broad-spectrum anthelmintic drug whose residual presence in food and the environment raises public health concerns, requiring rapid and sensitive methods of detection. In this work, a sensitive Fabry–Pérot interferometer (FPI) probe was fabricated by realizing a cavity located at the tip of a single-mode optical fiber core with a molecularly imprinted polymer (MIP) for ABZ detection. The fabrication process involved the development of a photoresist-based micro-hole filled by the specific MIP via thermal polymerization. Interferometric measurements obtained using the proposed sensor system have demonstrated a limit of detection (LOD) of 27 nM, a dynamic concentration range spanning from 27 nM (LOD) to 250 nM, and a linear response at the nanomolar level (27 nM–100 nM). The selectivity test demonstrated no signal when interfering molecules were present, and the application of the sensor for ABZ quantification in a commercial pharmaceutical sample provided good recovery, in accordance with bioanalytical validation standard methods. These results demonstrate the capability of a MIP layer-based FPI probe to provide low-cost and selective optical-sensing strategies, proposing a competitive approach to traditional analytical techniques for ABZ monitoring.

## 1. Introduction

Sensors can be classified by the type of transduction method employed. According to their sensing principle, sensors can be divided into physical sensors, chemical sensors, and biosensors [1,2,3]. Optical fiber sensors can be classified as physical sensors, as they rely on optical transduction mechanisms. However, when an optical transducer is combined with a chemically selective recognition layer, such as a molecularly imprinted polymer (MIP), the system can be defined as an optical-chemical sensor. In such sensors, the chemical binding event produces a physical change (e.g., variation in refractive index (RI)) that is optically measured by the transducer. In this work, we developed an interferometric optical-chemical sensor based on a Fabry–Pérot cavity, using an MIP specific for albendazole (ABZ), which combines the selectivity of MIPs with the high sensitivity and simplicity of a Fabry–Pérot interferometer (FPI).

Fiber optic sensors have been widely developed using various technologies, including grating-based devices and interferometers.

Nevertheless, despite their multiplexing capabilities, grating-based devices suffer from high fabrication costs and require specialized grating inscription equipment and personnel. On the other hand, optical fiber interferometers are easily fabricated through simple fiber post-processing techniques. Among the various fiber optic interferometers, the FPI has been the most widely reported thanks to its high sensitivity, good precision, and versatility [4]. This interferometer relies on two in-line fiber reflectors separated by a distance of a few microns. Light traveling across these devices is reflected at each of the reflectors, and due to the phase difference between the two reflected optical signals, when combined, they produce an interference fringe spectrum with maxima and minima, corresponding to constructive and destructive interference. The spectrum can therefore exhibit a wavelength shift or intensity change through changes in the medium between the reflectors (optical path length) or in the surrounding medium. Monitoring one of these variables makes it possible to obtain a relationship between those spectral changes and the variable under measurement.

FPI sensors have been realized by employing various technologies. Examples include a hollow silica tube between two single-mode fibers [5]; hollow cavities created by chemical etching [6]; and employing laser ablation techniques, such as femtosecond or excimer lasers [7]. Each of these approaches can involve challenging fabrication processes, including splicing special fibers, handling hazardous chemicals, or using costly laser technologies. On the other hand, FPI cavity fabrication through photopolymerizable resins has been one of the most straightforward and inexpensive methods. The simple fabrication involves hardening the resin found between two in-line fiber terminals [8,9]. The simplicity and versatility of these sensors led them to be employed in various applications, including strain, temperature, pressure, humidity, and curvature [8,9,10]. FPIs can also detect small RI variations in the media [1,8,9]. Thus, they have been gaining momentum in research applications related to the detection of substances for chemistry and biomedicine [11,12]. With their widespread deployment, they are nowadays commercially available for markets spanning biological sciences to medical applications [13].

In this context, there is a growing interest in sensing strategies that exploit synthetic receptors, particularly MIPs, which offer an interesting substitute to biological receptors like antibodies owing to their higher chemical and physical stability and their capability to function under extreme environmental situations [14,15,16]. Unlike antibodies, MIPs remain stable across a wide range of temperatures, pH values, and solvent conditions that would generally denature or degrade proteins, thereby providing an extended shelf life. MIP synthesis is generally cheaper than antibody production, and they can be manufactured on a wide scale with elevated reproducibility. Synthesized for a specific analyte, MIPs exhibit strong selectivity even when structurally related compounds are present. Furthermore, they can be regenerated multiple times cycles without losses in performances.

Due to MIPs’ lack of biological components, they minimize the risk of contamination and undesired immune responses [16,17,18]. Owing to these characteristics, MIPs have been incorporated in several platforms, including sensors, for applications in several fields, such as pharmaceutical analysis, medical diagnostics, and environmental monitoring [19,20,21].

MIPs combined with sensor technologies provide a highly sensitive and selective solution for the specific detection of a target analyte. Indeed, chemical sensors are able to monitor the presence and concentration variations in target analytes through physical or chemical sensing, such as electrical responses, optical variations, or color changes [22].

In particular, optical-chemical sensors translate the binding event between analyte and MIP into a measurable optical change, such as light absorption, fluorescence, light scattering, RI, or reflection [22]. Consequently, a range of optical techniques, including Ultraviolet/Visible spectroscopy (UV/Vis), fluorescence, chemiluminescence, Surface Plasmon Resonance (SPR), Raman scattering (RS), and FPI sensor, can be employed for MIP-based sensing [23,24].

The integration of MIPs with optical transducers thus offers a versatile, low-cost, and real-time solution for quantitative analyte monitoring in complex matrices.

In this study, an interferometric optical-chemical sensor based on a Fabry–Pérot cavity was developed. Specifically, the cavity was obtained at the tip of a single-mode optical fiber core by depositing an MIP for ABZ detection as a proof-of-concept.

ABZ is a broad-spectrum anthelmintic drug belonging to the benzimidazole class, widely used for treating parasitic infections in both humans and animals [25]. Its widespread use in veterinary medicine, combined with the possibility of residual accumulation in animal-derived food products and in the environment, makes it essential to develop rapid and sensitive methods for its monitoring [26].

Several analytical techniques have been reported for the determination of ABZ, including high-performance liquid chromatography (HPLC) [27,28,29,30], spectrophotometry [31,32,33], capillary electrophoresis [34,35], and titrimetric methods [36,37].

Electroanalytical approaches, such as voltammetry, have also been employed based on the oxidation of the drug at different electrode surfaces, including the hanging mercury drop electrode [38], glassy carbon electrodes [39,40], and a cathodically pretreated boron-doped diamond (BDD) electrode [41].

However, these traditional analytical techniques are often expensive, require long analysis times, and specialized personnel [42].

In this context, the combination of MIPs with sensors enables the development of selective, portable, and low-cost platforms useful for applications in the pharmaceutical, environmental, and food sectors, responding to the needs of food safety, environmental protection, and therapeutic monitoring [22].

Several ABZ sensors and biosensors have been reported, mainly based on electrochemical or fluorescence transductions [43,44,45,46]. No studies described MIP-based optical sensors, so we think there is room for introducing a new strategy for ABZ detection using a highly sensitive, selective, low-cost, and simple-to-realize system.

## 2. Materials and Methods

### 2.1. Chemicals and Reagents

Albendazole (ABZ, analytical standard, ≥98%), Glucose (GLU, ≥99.5%), Lactose (LACT, ACS reagent grade), Irbesartan (IBR, ≥98%), methacrylic acid (MAA, stabilized with hydroquinone monomethyl ether), divinylbenzene (DVB, stabilized with 4-tert-butylpyrocatechol), 2,2’-azo-bis-isobutyronitrile (AIBN, purum, ≥98.0%), and dimethyl sulfoxide (DMSO, HPLC grade solvent) were purchased from Merk Life Science S.r.l. (Milan, Italy). MAA and DVB were purified via filtration on an alumina column before use to remove stabilizers. All other reagents were used as received without further purification. Microposit S1813 photoresist was purchased from MicroChem Corp., (Westborough, MA, USA), while the developer solution (PMMA ARP 679.04) was from Allresist GmbH (Strausberg, Germany).

### 2.2. Real Sample

A tablet of the commercial drug Zentel, containing 400 mg of ABZ as the active principle, was dissolved in 4 mL of ultrapure water. Considering the molecular weight of ABZ (265.33 g/mol), the theoretical concentration of the resulting stock solution was approximately 0.38 M.

### 2.3. Preparation of the MIP Prepolymeric Solution

The MIP prepolymeric mixture was achieved by adapting a previously proposed procedure and using a classical molar ratio between the template, functional monomer, and cross-linker, i.e., 1:4:20 [47]. First, 20 mg of ABZ (template) were dissolved in 2 mL of DMSO, resulting in a 10 mg/mL solution. To 1 mL of the ABZ solution, 0.11 mL of MAA (functional monomer), 0.013 mL of DVB (cross-linker), and 20 mg of AIBN (radical initiator) were added, obtaining a molar ratio ABZ:MAA:DVB = 1:4:20. The solution was mixed and homogenized in an ultrasound bath for 15 min and then deoxygenated under a gentle flow of N_2_ for 10 min.

### 2.4. Sensing Principle and Optical Interferometry Background

**Figure 1:**
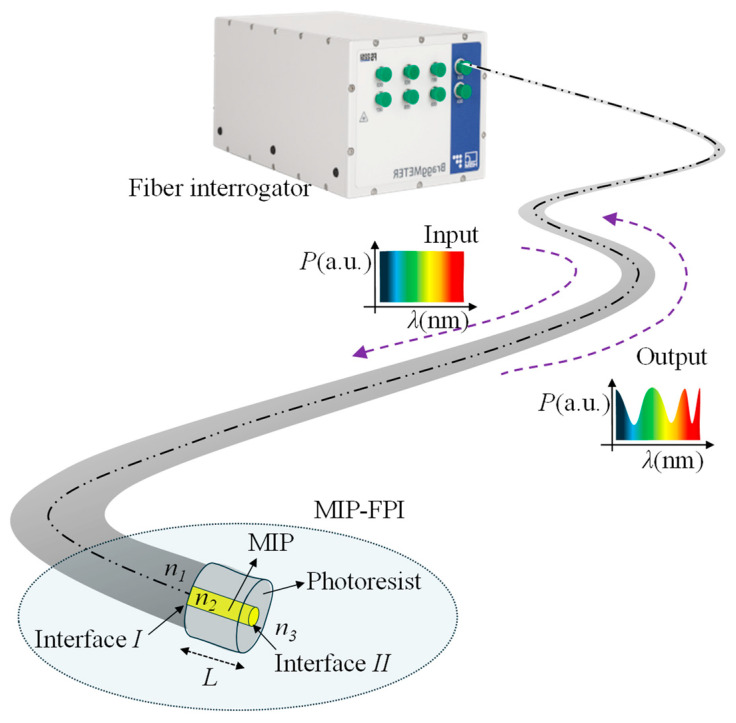
Schematic of the FPI measurement scheme, showing the light transmitted through the fiber and the resultant interference signal after the reflection.

In Figure 1, the interrogation scheme of an FPI fabricated at the tip of an optical fiber is shown. In this scheme, the interrogator injects a broadband optical signal into the optical fiber. At the far end of the fiber, the light beam will find two interfaces (*I* and *II*), specifically the one between *n*_1_ and *n*_2_ and the one between *n*_2_ and *n*_3_, respectively. Considering normal incidence, the Fresnel reflection coefficient at each of the interfaces can be calculated as follows:

(1) $$R_{1}=\;{\left|\frac{n_{1}-n_{2}}{n_{1}+n_{2}}\right|}^{2},\;R_{2}=\;{\left|\frac{n_{2}-n_{3}}{n_{2}+n_{3}}\right|}^{2}$$

where *n*_1_, *n*_2_, and *n*_3_ represent the RIs of the fiber core, MIP, and medium under analysis, respectively, as reported in Figure 1.

When the two reflections are recombined in the fiber core, they result in an interference fringe pattern at the output signal. Considering that the intensities of the reflected beams by the interfaces *I* and *II* are *I*_1_ and *I*_2_, respectively, the interference signal intensity can be written as

(2) $$I=I_{1}+I_{2}+2\sqrt{I_{1}I_{2}}cos\;\frac{2\pi }{\lambda }OPD$$

where *λ* is the free space wavelength and *OPD* is the optical path difference, which is given by *OPD* = 2*n*_2_L, with *n*_2_ and L being the cavity RI and length, respectively. The proposed sensing principle exploits the change in the MIP’s effective RI due to the binding with the analyte. Therefore, the sensor sensitivity will remain the same regardless of the sensor length.

### 2.5. Interferometric Measurements and Processing of Data

The interferometric measurements were performed by incubating the optical fiber tip, modified with the specific cavity filled with MIP, with aqueous solutions at increasing concentrations of ABZ. Subsequently, washing steps with ultrapure water were performed to remove any non-specific binding, and then the interferometric spectra were recorded with ultrapure water (blank) as bulk [48]. Variations in the interferometric spectrum were considered with respect to the blank (ultrapure water), in terms of wavelength (*Δλ*), for all the tested ABZ concentrations. The experimental data obtained from the binding tests were processed via Matlab software (vers. R2022b, Mathworks, Natick, MA, USA). Moreover, OriginPro 9 program (Origin Lab. Corp., Northampton, MA, USA) was employed to fit the experimental values via the Langmuir model, reported in Equation (3), in order to achieve the dose–response curve.

(3) $$\left|\Delta \lambda_{c}\right|=\left|\lambda_{c}-\lambda_{0}\right|=\left|\Delta \lambda_{\max}\right|\cdot \left(\frac{K_{\mathrm{aff}}\;\cdot \;c}{1+K_{\mathrm{aff}}\;\cdot \;c}\right)$$

In particular, *c* is the ABZ concentration, *Δλ_c_* is the wavelength variation registered for an ABZ solution at concentration *c*, *λ_0_* is the wavelength for the blank solution (water solution without ABZ), *Δλ_max_* is the maximum value of *Δλ_c_* attained at the saturation of the MIP’s sites with ABZ, and *K_aff_* is the MIP affinity constant for the specific analyte.

At low levels of ABZ concentrations, when $K_{\mathrm{aff}}\;\cdot \;c$ is considerably smaller than 1, Equation (3) can be approximated to a linear equation, reported in (4), the slope of which $\left|\Delta \lambda_{\max}\right|\cdot K_{\mathrm{aff}}$, is called sensitivity at low concentrations (*S*_low-conc_).

(4) $${|\Delta \lambda }_{c}|=\left|\Delta \lambda_{\max}\right|\cdot K_{\mathrm{aff}}\cdot c$$

From the linearization at low concentrations (Equation (4)), the limit of detection (LOD) can be estimated as the ratio between the standard deviation of replicates of blank solution measurements (*λ_0_*), multiplied through 3.3, and the *S*_low-conc_ [49].

## 3. MIP-Based Fabry–Pérot Optical Fiber Sensor

### 3.1. Experimental Setup

**Figure 2:**
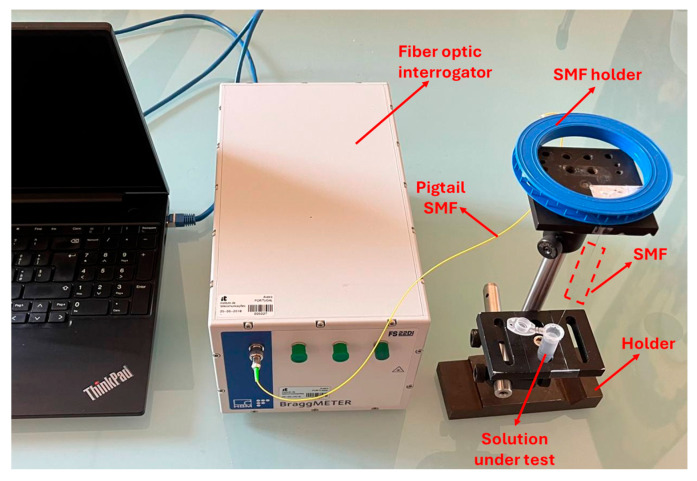
Picture of the experimental setup consisting of an optical interrogation system and optical fiber sensor connected onto it. The SMF tip was submerged in the liquid solution to be analyzed, with the data acquired and displayed on a PC.

Figure 2 shows the experimental setup used to check the interferometric signal, which includes a fiber optic interrogator, Industrial Bragg meter FS22 (from HBM FiberSensing SA, Maia, Portugal), with a maximum wavelength resolution of 5 pm. The optical-chemical sensor was based on a modified optical fiber. In particular, a single-mode fiber (SMF) was used, reference ITU G.652 (distributed by Cabelte SA, Vila Nova de Gaia, Portugal), with a core of 8.2 μm and a cladding of 125 μm. The SMF was secured in an SMF holder and connected to the fiber optic interrogator via the pigtail, as shown in Figure 2. Specifically, the fiber tip was immersed in the solution to be tested using a specific holder.

### 3.2. Production Steps of the Optical-Chemical Sensor

**Figure 3:**
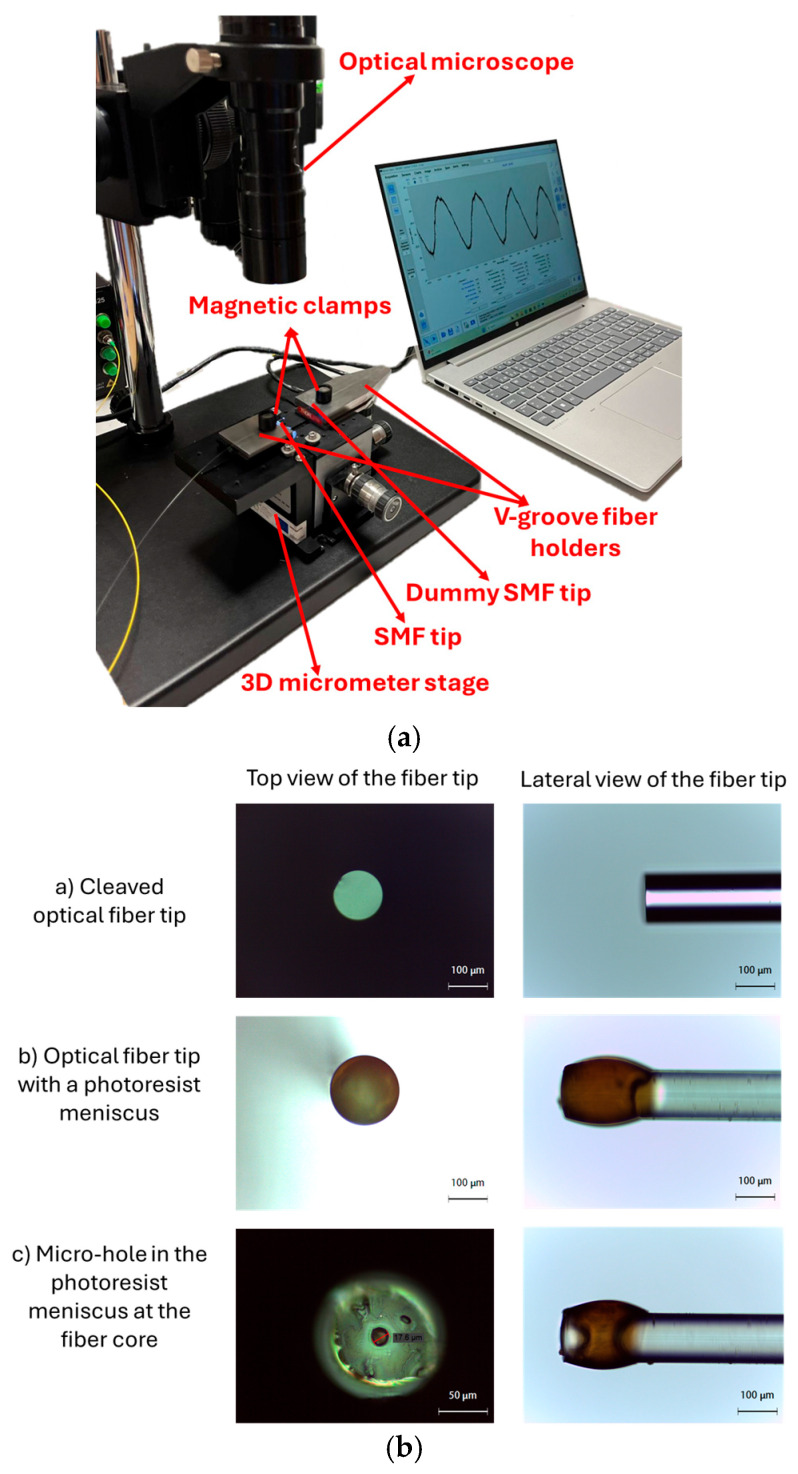
Production process of the optical-chemical sensor: (**a**) Experimental system description used for the dip-coating processes; (**b**) Sequence of images acquired via optical microscope showing the several steps in the optical sensor manufacturing process, from both top and lateral views of the fiber tip.

To realize the specific ABZ optical-chemical sensor, the SMF tip was first cleaved by using a Fujikura CT-30A fiber cleaver (Fujikura, Tokyo, Japan). The cleaved SMF was then dip-coated with S1813 photoresist via a bespoke dip-coater, as reported in Figure 3a. This setup includes an optical microscope for observing and aligning the fiber tips (one from the sensor and the other from a dummy single-mode fiber, or SMF), which are secured by V-groove fiber holders and magnetic clamps, all mounted on a three-axis micrometer stage that enables high-precision movements. The SMF tip was positioned and controlled in real time, while a computer acquires the real-time image from the microscope and the optical signal from the interrogator during the coating process. Specifically, the dummy fiber had photoresist on its tip, and the prepared SMF tip of the sensor was precisely moved in the direction of the dummy fiber to touch the resist found on it and to then return to its original position, forming a concave meniscus on its tip.

The obtained photoresist-based meniscus on the fiber tip was thermally polymerized in an oven at 80 °C for 15 min. Subsequently, the pigtail fiber was connected to a 405 nm laser source (a laser power) and irradiated for 1 s. The fiber tip was then observed under an optical microscope (in both lateral and top views of the fiber tip). The irradiated area was developed using the appropriate solution, after which the fiber tip was re-examined under the microscope to verify the presence of a micro-hole (approximately 20–50 µm in total diameter) at the fiber center (core region) and to assess any potential side effects of the developer on the non-irradiated resist regions.

Figure 3b shows the images acquired via optical microscope after each step of the described optical sensor fabrication process.

Finally, the modified fiber tip was dip-coated with the MIP prepolymeric mixture and placed in a thermostatic oven overnight at 75 °C for thermal polymerization. The template and unreacted monomers were subsequently removed through washing steps with isopropanol, followed by rinsing with ultrapure water. After the polymerization and washing steps, the MIP layer is stable, and no loss of material was evidenced during the measurements for the whole dose–response curve.

During sensor processing development, the reflected spectrum can be monitored to obtain information about the core of the fiber tip. In particular, after the cleaving process, the spectrum exhibited a flat optical power response across the wavelength, a consequence of Fresnel reflection at the fiber tip. Then, the photoresist (S1813) deposition can be monitored by an interference fringe signal. Later, after the developing stage, the core tip region was clean, and the reflected spectrum again had a flat response. Finally, after the MIP deposition (the hollow region left in the photoresist is filled with the MIP), a reflected spectrum appears with an interference fringe pattern originating from the MIP-based FPI cavity on the fiber tip.

## 4. Results and Discussion

### 4.1. Binding Tests

**Figure 4:**
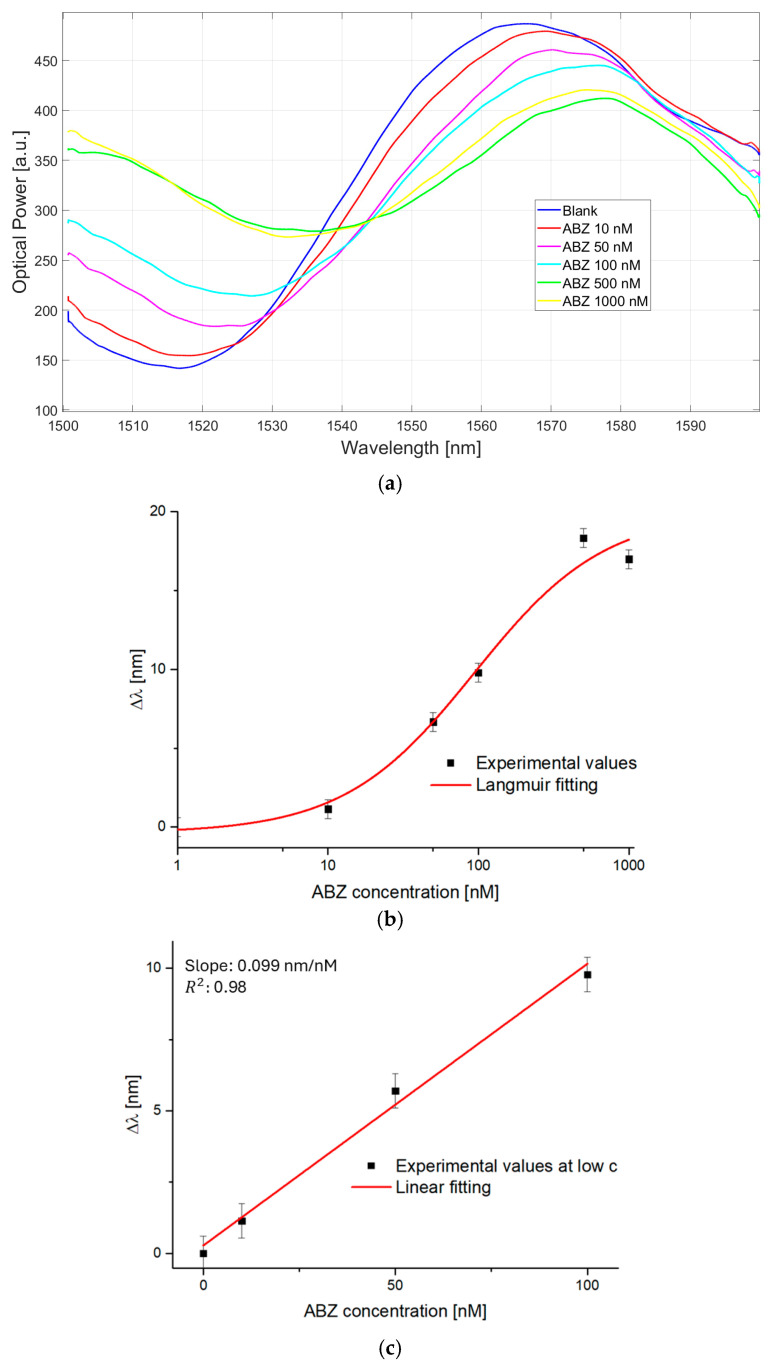
Results of the binding tests performed with the MIP-based Fabry–Pérot optical fiber sensor at different ABZ standard solutions: (**a**) Interferometric spectra obtained with the optical-chemical sensors at increasing ABZ concentration; (**b**) Dose–response plot in semilogarithmic scale achieved by monitoring the spectral shifts, with error bars; The data fitting attained via Equation (3) is reported as a solid red line; (**c**) Linear response achieved at low ABZ concentrations, with error bars.

The interferometric spectra, recorded by testing the MIP-based Fabry–Pérot optical fiber sensor with different ABZ standard solutions (from 10 to 1000 nM), according to the protocol described in Section 2.5, are shown in Figure 4a. Usually, the binding analyte-MIP produces a variation in the RI of the MIP receptor layer; thus, a wavelength shift with the ABZ concentration happens [48]. More specifically, these bindings lead to an increase in the RI of the MIP layer located at the tip of the optical fiber, thus resulting in the wavelength red-shift [48]. In fact, in this case, the combination of the optical fiber sensor with the specific MIP for ABZ detection induces a wavelength shift towards higher values (red-shift) by increasing the ABZ concentration, as reported in Figure 4a. This result indicated that when the binding of ABZ-MIP occurs, the MIP RI value increases [48]. In Figure 4b, the dose–response curve is illustrated on a semilogarithmic scale. This curve was obtained by reporting the specific optical signal change (*Δλ*), estimated in relation to the blank (water solution free of ABZ), as a function of the ABZ concentrations. It is important to highlight that the whole curve was only to be considered for the Langmuir parameters determination, which is useful to achieve the chemical sensor parameters. The fitting parameters resulting from the Langmuir model and the sensor’s analytical figures of merit (sensitivity and LOD) are reported in Table 1. On the other hand, only the dynamic concentration range should be used for quantitative analysis, i.e., the range spanning from 27 nM (LOD) to 250 nM, before the curve reaches its plateau. In order to highlight the linear range of the sensor, Figure 4c shows the linear fitting of the experimental data obtained at low ABZ concentrations.

The data, reported in both Figure 4b,c as black squares, represent the average value of the measurements achieved with three analogous optical-chemical sensors, which were evaluated under comparable conditions. Finally, the error bars indicate the standard deviation of these tests.

### 4.2. Selectivity Tests

**Figure 5:**
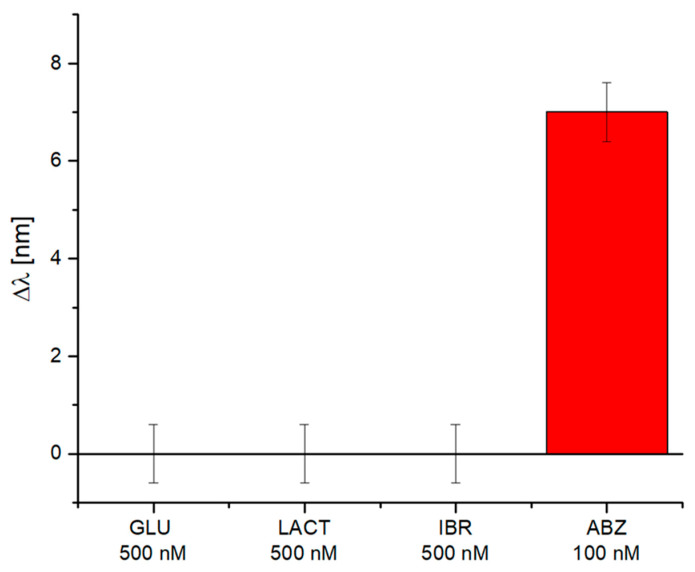
Selectivity test results: the MIP-based Fabry–Pérot optical fiber sensor tested with several interfering molecules (GLU, LACT, and IBR) in aqueous solutions at a concentration of 500 nM each. The sensor signal (*Δλ*) was compared with that obtained by testing the target analyte (ABZ) at 100 nM.

After testing the MIP-based Fabry–Pérot optical fiber sensor for ABZ detection in water, further tests were made to evaluate the MIP selectivity. Specifically, Glucose (GLU), Lactose (LACT), and Irbesartan (IBR) were tested, due to their presence in pharmaceutical formulations [50,51,52]. In particular, the optical-chemical sensor was tested with 500 nM of each interfering molecule. However, despite these high concentration levels of potential interferents, the MIP-based Fabry–Pérot optical fiber sensor did not register any response, as shown in Figure 5. Instead, the optical-chemical sensor recorded a significant response (*Δλ*) only after incubation with the target analyte (ABZ) at a lower concentration, of approximately 100 nM, as shown in Figure 5. Moreover, the obtained value did not differ from that reported in the dose–response curve (Figure 4b).

This result highlights the selectivity of MIP-based Fabry–Pérot interferometer probe for ABZ monitoring.

### 4.3. Real Sample Analysis: Evaluation of ABZ in Pharmaceutical Formulation

The real sample stock solution, prepared as described in Section 2.2, was diluted with ultrapure water to obtain diluted solutions at 1:5·10^6^ and 1:2.5·10^6^, corresponding to expected ABZ concentrations of about 75 nM and 150 nM, respectively. Each diluted solution was tested using the developed MIP-based Fabry–Pérot optical fiber sensor via the same experimental protocol applied for the dose–response curve (Section 2.5). The sensor signals were converted to concentration values using the calibration curve previously obtained with standard solutions (Figure 4b), which were multiplied by the corresponding dilution factors to estimate the ABZ concentration in the stock solution, as reported in Table 2. The values obtained corresponded to 0.3 M (Table 2), which represents ~80% recovery compared to the theoretical concentration (0.38 M).

Considering the poor aqueous solubility of ABZ and the presence of excipients in the commercial formulation, this recovery is consistent with expectations and falls within the commonly accepted range (80–120%) for bioanalytical validation [53].

### 4.4. Comparative Analysis of ABZ Sensors

From the tests carried out, the developed optical-chemical sensor shows high sensitivity and specificity in the detection of ABZ at the nanomolar level. Compared to most sensors and bio/chemical sensors reported in the literature, the MIP-based Fabry–Pérot optical fiber sensor shows a lower detection range, comparable only to two electrochemical sensors [45,54], as reported in Table 3.

Despite the good sensitivity of the two electrochemical sensors listed in Table 3 [45,54], they have some limitations. In fact, the reproducibility of disposable electrodes is often limited, requiring laborious surface pre-treatment and leading to batch-to-batch variability [45]. Furthermore, electrode fouling and interference from electroactive species in complex matrices can compromise selectivity and long-term performance.

The used nanomaterials, such as MXenes, also present oxidation and stability issues that limit their shelf life [54]. Furthermore, electrochemical systems typically require dedicated potentiostats and stable reference electrodes, which complicate their integration into portable or on-site devices.

In contrast, the developed optical-chemical sensor is based on MIPs, which provides highly selective binding sites for ABZ, as demonstrated in both Section 4.3 and Section 4.4. Moreover, optical interrogation of the Fabry–Pérot cavity enables label-free detection without the need for electrochemical instrumentation.

Furthermore, the polymeric nature of MIPs ensures chemical robustness and long-term stability. These characteristics highlight the potential of the proposed optical-chemical sensor as a competitive and advantageous alternative to state-of-the-art electrochemical sensors for ABZ detection.

In other words, the proposed optical-chemical sensor utilizes the MIP layer, thereby exhibiting high chemical and physical stability. In fact, MIPs are well-known to operate reliably even under extreme environmental conditions. MIPs are also known for their long lifetime, retaining repeatability, and the ability to be reused multiple times following regeneration processes [14,15,16].

## 5. Conclusions

In this work, for the first time, a Fabry–Pérot interferometer was realized via molecularly imprinted polymers on the core of an optical fiber tip. As a proof of concept, an MIP for the detection of albendazole was used. The proposed optical-chemical sensor demonstrated high sensitivity at the nanomolar level and selectivity towards the target analyte. Tests were also conducted on a real pharmaceutical sample to demonstrate the sensor’s capability in a complex matrix. Compared to other sensing approaches, the proposed sensor system offers advantages in terms of the stability of the MIP and potential miniaturization, while retaining all the inherent characteristics of the optical fiber probes.

More specifically, this work demonstrated that integrating MIPs with optical transducers paves the way for the development of robust and sensitive analytical probes for food safety applications, environmental monitoring, and pharmaceutical control. It is essential to emphasize that, conventionally, Fabry–Pérot interferometers have been designed and developed to measure physical quantities, whereas their application in chemical sensing remains limited [8,9,10,11,12,13]. In this study, for the first time, a Fabry–Pérot cavity has been formed using an MIP layer, thereby integrating the recognition and transduction functions within a single optical cavity. This approach combines the high sensitivity of interferometric transduction with the chemical selectivity and robustness of MIPs. Compared to previously reported electrochemical or fluorescence-based ABZ sensors, the proposed device offers label-free detection, simple and low-cost fabrication, and high selectivity, while maintaining a nanomolar level. Therefore, the main advantages of the developed sensor are its high sensitivity, low-cost, simple manufacturing process, and excellent chemical stability. However, the system still presents some limitations, mainly related to the current stage of miniaturization, which does not yet allow for fully portable, on-site ABZ measurements.

## Figures and Tables

**Table 1:** Fitting parameters derived from the Langmuir model and sensor’s analytical figures of merit obtained via the MIP-based Fabry-Pérot interferometer probe for ABZ detection. The numbers in parentheses indicate the standard deviation of the last digit.

*λ_0_* [nm]	*Δλ_max_* [nm]	*K_aff_* [nM]^−1^	R^2^	*S*_low-conc_	LOD [nM]
−1.19(2)	19.8(1.5)	0.012(5)	0.97	0.25 nm/nM	27

**Table 2:** Estimation of ABZ concentration in diluted commercial pharmaceutical samples.

Sample	*Δλ* [nm]	ABZ Concentration in the Diluted Samples	Estimated ABZ Concentration
Real sample diluted 1:5 × 10^6^	7.4	58 nM	58 nM × 5·10^6^ = 0.29 M
Real sample diluted 1:2.5 × 10^6^	11	116 nM	116 nM × 2.5·10^6^ = 0.3 M

**Table 3:** Comparative analysis of several sensors and bio/chemical sensors developed for ABZ detection exploiting different sensing principles and/or receptors.

Sensor	Sensing Principle	Type of Receptor	Detection Range	LOD	Reference
Biopolymer-derived nanoconfiguration	Electrochemical	MIP	Micromolar	0.45 μM	[43]
Pretreated Graphite Pencil Electrode	Electrochemical	-	Nanomolar	5.42 nM	[45]
Pt-Pd-Modified Glassy Carbon Electrode	Electrochemical	-	Micromolar	0.08 μM	[44]
Integrated three-electrode devices based on alkalized Ti3C2Tx/LIG	Electrochemical	-	Nanomolar	7.5 nM	[54]
Dual-emissive Eu(III)-functionalized metal–organic frameworks	Fluorescence	-	Micromolar	0.1 μM	[46]
Nanoaptasensor based on AuNTs	Plasmonic	Aptamer	Micromolar	13 nM	[55]
MIP-based Fabry-Pérot optical fiber probe	Interferometric	MIP	Nanomolar	27 nM	This work

## Data Availability

Data will be made available upon request.

## References

[1] Frazão O., Baptista J.M., Santos J.L., Kobelke J., Schuster K. (2009). Refractive index tip sensor based on Fabry-Pérot cavities formed by a suspended core fibre. J. Eur. Opt. Soc.-Rapid Publ..

[2] Li J.H., Wu J., Yu Y.X. (2021). DFT exploration of sensor performances of two-dimensional WO3 to ten small gases in terms of work function and band gap ghanges and I-V responses. Appl. Surf. Sci..

[3] Kao K., Alocilja E.C. (2025). Integrated sample to detection of carbapenem-resistant bacteria extracted from water samples using a portable gold nanoparticle-based biosensor. Sensors.

[4] Rao Y.J., Ran Z.L., Gong Y. (2017). Fiber-Optic Fabry–Pérot Sensors: An Introduction.

[5] Choi H.Y., Park K.S., Park S.J., Paek U.C., Lee B.H., Choi E.S. (2008). Miniature Fiber-Optic high temperature sensor based on a hybrid structured Fabry–Pérot interferometer. Opt. Lett..

[6] Tafulo P.A.R., Jorge P.A.S., Santos J.L., Araujo F.M., Frazao O. (2012). Intrinsic Fabry–Pérot cavity sensor based on etched multimode graded index fiber for strain and temperature measurement. IEEE Sens. J..

[7] Liao C.R., Hu T.Y., Wang D.N. (2012). Optical fiber Fabry-Pérot interferometer cavity fabricated by femtosecond laser micromachining and fusion splicing for refractive index sensing. Opt. Express.

[8] Oliveira R., Bilro L., Nogueira R. (2018). Fabry-Pérot cavities based on photopolymerizable resins for sensing applications. Opt. Mater. Express.

[9] Oliveira R., Bilro L., Marques T.H.R., Cordeiro C.M.B., Nogueira R. (2019). Simultaneous detection of humidity and temperature through an adhesive based Fabry–Pérot cavity combined with polymer fiber bragg grating. Opt. Lasers Eng..

[10] Oliveira R., Cardoso M., Rocha A.M. (2022). Two-dimensional vector bending sensor based on Fabry-Pérot cavities in a multicore Fiber. Opt. Express.

[11] Zhao Y., Li C., Lin Z., Wang Y., Tong R., Cai L. (2024). Plug-and-play Fabry-Pérot interferometric biosensor with vernier effect for label-free detection of bovine serum albumin. Sens. Actuators B Chem..

[12] Qiu H., Yao Y., Dong Y., Tian J. (2024). Fiber-optic immunosensor based on a Fabry–Pérot interferometer for single-molecule detection of biomarkers. Biosens. Bioelectron..

[13] FISO Technologies Inc.. http://www.fiso.com.

[14] Haupt K., Linares A.V., Bompart M., Bui B.T.S. (2011). Molecularly imprinted polymers. Topics in Current Chemistry.

[15] BelBruno J.J. (2018). Molecularly imprinted polymers. Chem. Rev..

[16] Chen L., Xu S., Li J. (2011). Recent Advances in molecular imprinting technology: Current status, challenges and highlighted applications. Chem. Soc. Rev..

[17] Vasapollo G., Del Sole R., Mergola L., Lazzoi M.R., Scardino A., Scorrano S., Mele G. (2011). Molecularly imprinted polymers: Present and future prospective. Int. J. Mol. Sci..

[18] Mahony J.O., Nolan K., Smyth M.R., Mizaikoff B. (2005). Molecularly imprinted polymers—Potential and challenges in analytical chemistry. Anal. Chim. Acta.

[19] Büyüktiryaki S. (2025). Molecularly imprinted polymer nanoparticles for pharmaceutical applications: Sample preparation, sensor-based detection, and controlled drug release. Polymers.

[20] Kadhem A.J., Gentile G.J., Fidalgo de Cortalezzi M.M. (2021). Molecularly imprinted polymers (MIPs) in sensors for environmental and biomedical applications: A review. Molecules.

[21] Rebelo P., Costa-Rama E., Seguro I., Pacheco J.G., Nouws H.P.A., Cordeiro M.N.D.S., Delerue-Matos C. (2021). Molecularly imprinted polymer-based electrochemical sensors for environmental analysis. Biosens. Bioelectron..

[22] Huang X., Xia L., Li G. (2023). Recent progress of molecularly imprinted optical sensors. Chemosensors.

[23] Henry O.Y.F., Cullen D.C., Piletsky S.A. (2005). Optical interrogation of molecularly imprinted polymers and development of MIP sensors: A Review. Anal. Bioanal. Chem..

[24] Arcadio F., Del Prete D., Zeni L., Cennamo N., Seggio M. (2025). Optical sensor chips monitored via extrinsic optical fiber schemes. IEEE Sens. Rev..

[25] Horton J. (2000). Albendazole: A Review of anthelmintic efficacy and safety in humans. Parasitology.

[26] Ido A., Inoue Y., Okujima C., Okamoto M., Miura T., Kawashima A., Takahashi S. (2025). Distribution and persistence of the anthelmintic drug albendazole in yellowtail aquaculture. Aquac. Rep..

[27] Kitzman D., Cheng K.-J., Fleckenstein L. (2002). HPLC Assay for albendazole and metabolites in human plasma for clinical pharmacokinetic studies. J. Pharm. Biomed. Anal..

[28] Valois M.E.C., Takayanagui O.M., Bonato P.S., Lanchote V.L., Carvalho D. (1994). Determination of albendazole metabolites in plasma by HPLC. J. Anal. Toxicol..

[29] Wu Z., Medlicott N.J., Razzak M., Tucker I.G. (2005). Development and optimization of a rapid HPLC method for analysis of ricobendazole and albendazole sulfone in sheep plasma. J. Pharm. Biomed. Anal..

[30] Melikyan S., Biront N., Venyatynska O., Pazderska O., Mysko G., Yanovych D. (2021). Development of methods for quantitative determination of albendazole and its metabolites in biological tissues using HPLC/FLD. Sci. Tech. Bull. State Sci. Res. Control. Inst. Vet. Med. Prod. Fodd. Addit. Inst. Anim. Biol..

[31] Refat M.S., Mohamed G.G., Fathi A. (2011). Spectrophotometric determination of albendazole drug in tablets: Spectroscopic characterization of the charge-transfer solid complexes. Chin. J. Chem..

[32] Ahmed D.A., Abdel-Aziz O., Abdel-Ghany M., Weshahy S.A. (2018). Stability indicating determination of albendazole in bulk drug and pharmaceutical dosage form by chromatographic and spectrophotometric methods. Future J. Pharm. Sci..

[33] Soto C., Contreras D., Orellana S., Yañez J., Toral M.I. (2010). Simultaneous determination of albendazole and praziquantel by second derivative spectrophotometry and multivariated calibration methods in veterinary pharmaceutical formulation. Anal. Sci..

[34] Paias F.O., Lanchote V.L., Takayanagui O.M., Bonato P.S. (2001). Enantioselective analysis of albendazole sulfoxide in cerebrospinal fluid by capillary electrophoresis. Electrophoresis.

[35] Prost F., Caslavska J., Thormann W. (2002). Chiral analysis of albendazole sulfoxide enantiomers in human plasma and saliva using capillary electrophoresis with on-column absorption and fluorescence detection. J. Sep. Sci..

[36] Basavaiah K., Prameela H.C. (2003). Two simple methods for the estimation of albendazole and its dosage forms using chloramine-T. Il Farm..

[37] Basavaiah K., Prameela H.C. (2003). Use of an oxidation reaction for the quantitative determination of albendazole with chloramine-T and acid dyes. Anal. Sci..

[38] Abu Zuhri A.Z., Hussein A.I., Musmar M., Yaish S. (1999). Adsorptive stripping voltammetric determination of albendazole at a hanging mercury drop electrode. Anal. Lett..

[39] Santos A.L., Takeuchi R.M., Mariotti M.P., De Oliveira M.F., Zanoni M.V.B., Stradiotto N.R. (2005). Study of electrochemical oxidation and determination of albendazole using a glassy carbon-rotating disk electrode. Il Farm..

[40] Gowda J.I., Kantikar R.B., Harakuni D.G., Jadhav K.Y., Chanagoudar V.C., Nandibewoor S.T. (2016). Electrochemical determination of albendazole at glassy carbon electrode. J. AOAC Int..

[41] Lourencao B.C., Baccarin M., Medeiros R.A., Rocha-Filho R.C., Fatibello-Filho O. (2013). Differential Pulse Voltammetric Determination of Albendazole in Pharmaceutical Tablets Using a Cathodically Pretreated Boron-Doped Diamond ElectrodeJ. Electroanal. Chem..

[42] Vázquez E.M., Romero B., Sahagún A.M., López C., de la Puente R., Rodríguez J.M., Fernández N., Diez M.J., Díez R. (2025). Analytical method for the simultaneous determination of albendazole and metabolites using HPLC-PDA: A validation study. Molecules.

[43] Srivastava J., Singh M. (2016). A biopolymeric nano-receptor for sensitive and selective recognition of albendazole. Anal. Methods.

[44] Suaifan G.A.R.Y., Khanfar M.F., Shehadeh M.B., Alnajajrah A., Abuhamdan R., Hasan S.A. (2022). An electrochemical sensor for the detection of albendazole using glassy carbon electrode modified with platinum-palladium nanocomposites. Biosensors.

[45] Gowda J.I., Hurakadli G.S., Nandibewoor S.T. (2017). Pretreated graphite pencil electrode based voltammetric sensing of albendazole. Anal. Chem. Lett..

[46] Yuan H.-Q., Xia Y.-F., Zhong Y.-F., Li W., Zhu H., Wang R., Chen P., Gao Z., Zhu X., Li Y.-X. (2024). Dual-emissive Eu(III)-functionalized metal-organic frameworks for visual, rapid, and intelligent sensing of albendazole and albendazole sulfoxide in animal-origin food. Anal. Chim. Acta.

[47] Cacho C., Turiel E., Pérez-Conde C. (2009). Molecularly imprinted polymers: An analytical tool for the determination of benzimidazole compounds in water samples. Talanta.

[48] Cennamo N., Massarotti D., Conte L., Zeni L. (2011). Low cost sensors based on SPR in a plastic optical fiber for biosensor implementation. Sensors.

[49] Desimoni E., Brunetti B. (2015). About estimating the limit of detection by the signal to noise approach. Pharm. Anal. Acta.

[50] Záhonyi P., Szabó E., Domokos A., Haraszti A., Gyürkés M., Moharos E., Nagy Z.K. (2022). Continuous integrated production of glucose granules with enhanced flowability and tabletability. Int. J. Pharm.

[51] Hebbink G.A., Dickhoff B.H.J. (2019). Application of Lactose in the Pharmaceutical Industry. Lactose.

[52] Gillis J.C., Markham A. (1997). Irbesartan A Review of its Pharmacodynamic and Pharmacokinetic Properties and Therapeutic Use in the Management of Hypertension. Drugs.

[53] Tiwari G., Tiwari R. (2010). Bioanalytical method validation: An updated review. Pharm. Methods.

[54] Zhou X., Liu M., Yang B., Wu C., Wu K., Sun S. (2025). Alkalized MXene/laser-induced graphene-based integrated three-electrode devices for micro-droplet detection of albendazole. Anal. Chim. Acta.

[55] Molina C.L., Puma M.C.L., Hernandez Y., Castillo J.E.F., Rimache J.R., Acosta J., Galarreta B.C., Eguiluz M. (2025). Study of the interaction of the aptamer Cz12 with albendazole sulfoxide by molecular docking and Uv-Vis-Nir spectroscopy. SSRN Prepr..

